# Supinated Foot Posture Is Associated With Reduced Knee and Foot Function in Patients With Knee Osteoarthritis

**DOI:** 10.7759/cureus.85201

**Published:** 2025-06-01

**Authors:** Shingo Mitamura, Akito Kataoka, Hideki Warashina

**Affiliations:** 1 Rehabilitation, Nagoya Joint Replacement and Orthopedic Clinic, Kitanagoya, JPN; 2 Orthopedic Surgery, Nagoya Joint Replacement and Orthopedic Clinic, Kitanagoya, JPN

**Keywords:** ankle dorsiflexion rom, femorotibial angle, foot posture index-6, isometric knee extension strength, knee flexion rom, toe grip strength

## Abstract

Objective: This study investigated the difference in lower limb function between supinated and neutral foot postures in patients with knee osteoarthritis (KOA) scheduled for total knee arthroplasty (TKA).

Methods: This study included 62 female patients with KOA knees. Patients were classified into the neutral foot posture group (n = 33 (53%)) and the supinated foot posture group (n = 29 (47%)) using the Foot Posture Index-6 (FPI-6). Femorotibial angle (FTA), knee flexion range of motion (ROM), ankle dorsiflexion ROM, knee extension strength, toe grip strength (TGS), Timed Up and Go (TUG) Test, and knee pain were compared between the two groups.

Results: The supinated foot posture group had significantly lower knee flexion ROM, ankle dorsiflexion ROM, knee extension strength, TGS, and larger FTA than those in the neutral foot posture group. However, no significant differences were observed in TUG or knee pain.

Conclusion: KOA patients with supinated feet have reduced lower limb strength and ROM, suggesting that incorporating foot alignment assessment into KOA rehabilitation is important. However, long-term studies are needed to clarify causal relationships.

## Introduction

Knee osteoarthritis (KOA) is a prevalent degenerative joint disease that leads to pain, functional limitations, and reduced quality of life. Lower limb alignment and foot posture play crucial roles in KOA progression by influencing biomechanical loading, joint stability, and gait mechanics [1,2]. The Foot Posture Index-6 (FPI-6) is commonly used to classify foot posture as supinated (≤ -1), neutral (0-5), or pronated (≥ +6) [3,4]. Among these, supinated foot posture has been associated with altered weight distribution, reduced shock absorption, and compensatory changes in knee joint mechanics [5].

Previous studies suggest that deviations from a neutral foot posture, particularly supination, may contribute to lower extremity functional impairments [6]. Supinated foot posture is also linked to reduced ankle dorsiflexion, which can exacerbate compensatory gait patterns and increase stress on the knee joint [7]. Furthermore, biomechanical changes such as increased knee adduction moment, associated with medial shift of the ground reaction force, are observed in foot supination, potentially promoting cartilage degeneration, joint space narrowing, and ultimately KOA progression [8,9]. Karataş et al. [9] further demonstrated that a higher medial longitudinal arch, typically seen in foot supination, is correlated with greater knee varus alignment and radiographic severity in KOA patients.

While these findings suggest that foot posture can influence KOA pathophysiology, the relationships between foot posture and specific functional outcomes, such as femorotibial angle (FTA), knee extension strength, and toe grip strength (TGS), remain unclear. 

This study aimed to determine whether patients with supinated foot posture (FPI-6 ≤ -1) exhibited differences in FTA, knee range of motion (ROM), isometric knee extension strength (IKES), TGS, and ankle dorsiflexion ROM compared to those with neutral foot posture (0 ≤ FPI-6 ≤ 5).

## Materials and methods

Ethical statement

This study was approved by the Institutional Review Board of the Nagoya Orthopedic Joint Replacement Clinic Ethics Committee (Approval No. 202403001) and was conducted in accordance with the principles outlined in the Declaration of Helsinki. All participants provided written informed consent prior to participation.

Study participants

**Figure 1:**
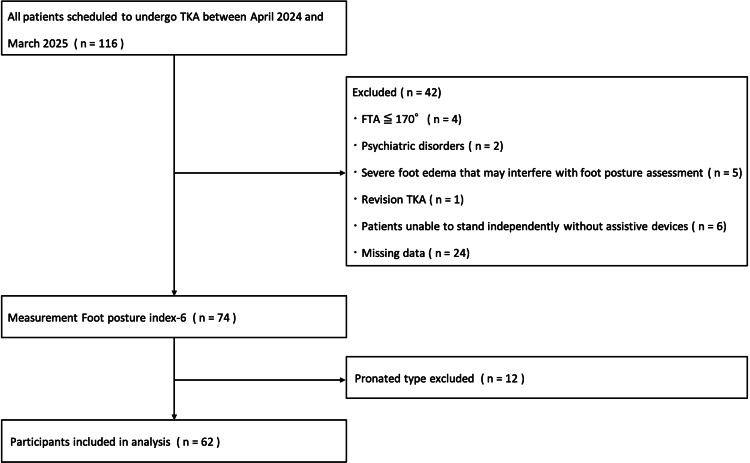
Flow diagram of the inclusion/exclusion criteria for the analysis TKA: total knee arthroplasty; FTA: femorotibial angle

The required sample size was determined based on a power analysis informed by previous studies investigating biomechanical differences associated with foot posture [10,11]. To detect a percentage difference between groups with a statistical power of 0.85, a minimum sample size of 58 patients (29 per group) was calculated. Additionally, for evaluating differences in continuous variables between unpaired groups, the calculated sample size was 46 patients (23 per group) to achieve a power of 0.90. This study enrolled 101 female patients (116 knees) diagnosed with KOA, who were scheduled for total knee arthroplasty (TKA) between April 2024 and March 2025. The inclusion criteria were (i) female patients aged 50-90 years and (ii) a confirmed clinical and radiological diagnosis of KOA with a Kellgren-Lawrence (KL) grade of ≥3. The decision to include only female patients was based on previous research: Uritani et al. [12] included exclusively female KOA patients, noting their predominance among clinical cases. Additionally, Uritani et al. [13] highlighted gender differences in TGS, further justifying our decision to focus exclusively on female participants to maintain homogeneity and enhance generalizability within this specific population. Exclusion criteria were (ⅰ) FTA ≤ 170°, (ⅱ) individuals with psychiatric disorders, (ⅲ) severe foot edema that may interfere with foot posture assessment, (ⅳ) patients undergoing revision TKA, (ⅴ) patients unable to stand independently without assistive devices, and (ⅵ) patients with missing assessment data. Participants' foot postures were classified using the FPI-6 into three categories: pronated, neutral, or supinated. However, the pronated foot group (n = 12) was excluded from the comparative analysis due to an insufficient number of participants, which would have precluded meaningful statistical comparisons. Therefore, analyses focused exclusively on comparisons between patients with supinated and neutral foot postures (Figure 1).

Clinical outcomes

Foot Posture Assessment

**Figure 2:**
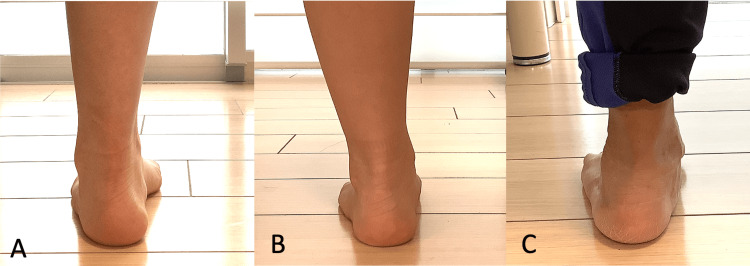
Representative photos of feet classified using the FPI-6 FPI-6: Foot Posture Index-6 Supinated foot posture (A): the calcaneus is inverted, the medial longitudinal arch is high, and the medial toes are clearly visible. Neutral foot posture (B): the calcaneus is vertical, the curve above and below the malleolus is identical, and the lateral and medial toes are equally visible. Pronated foot posture (C): the calcaneus is everted, the medial longitudinal arch is in contact with the ground, and the lateral toes are clearly visible

FPI-6 was used to classify the foot posture of participants into pronated, neutral, or supinated categories according to previously validated criteria [14]. During the assessment, participants stood barefoot in a relaxed, weight-bearing stance with equal distribution on both feet. One trained examiner conducted evaluations on the foot scheduled for TKA using the following six clinical criteria: (ⅰ) the prominence of the talar head, palpated medially or laterally; (ⅱ) the curvature observed above and below the lateral malleolus, indicating the degree of supra- and inframalleolar curvature; (ⅲ) frontal-plane alignment of the calcaneus, classified as everted, neutral, or inverted; (ⅳ) prominence around the talonavicular joint region, specifically noting any medial bulging of the navicular bone; (ⅴ) congruence and shape of the medial longitudinal arch, assessing its height and contour; and (ⅵ) the relative positioning of the forefoot to the rearfoot, noting whether it is abducted or adducted. Each criterion was individually scored from -2 (indicating highly supinated posture) to +2 (indicating highly pronated posture). The sum of these six items was then used to determine overall foot posture classification as follows: pronated foot (+6 to +12), neutral foot (0 to +5), or supinated foot (-1 to -12) (Figure 2).

Toe Grip Strength (TGS) 

TGS was assessed using a toe grip dynamometer (T.K.K. 3362, Takei Scientific Instrument Co., Niigata, Japan) [15]. Participants were seated in an upright position with their hips and knees flexed at 90° and their ankles positioned in a neutral alignment. To ensure a stable posture and minimize extraneous movements, they maintained their arms crossed over their chest throughout the measurement. The affected foot was placed on the dynamometer, and the foot was fixed with a belt. Participants were instructed to use their toes to grip the bar with maximum force for at least three seconds. Sufficient practice was performed to ensure that maximum muscle strength could be measured. Each participant performed two trials, and the maximum muscle strength reached during the two trials was normalized to the participant’s body weight. The normalized values were used for data analysis.

Knee Alignment

The FTA was measured using standing full-length lower limb anteroposterior radiographs taken within six months before surgery.

Range of Motion (ROM) of the Knee and Ankle and Standing Knee Extension Angle

Knee flexion and extension ranges were measured at the intersection of the mechanical axis of the femur (a line from the greater trochanter to the lateral epicondyle of the femur) and the lower leg (a line from the head of the fibula to the lateral malleolus of the fibula). Passive knee flexion and extension ranges were measured using a standard goniometer with the participant lying on her back. In addition, extension angles were measured with the participant standing. The participant was instructed to be barefoot and to assume her usual standing position. The range of ankle dorsiflexion was measured at the intersection of a perpendicular line to the long axis of the fibula in the sagittal plane with the plantar surface of the foot. This was measured using a standard goniometer with the subject lying supine with the knee extended.

Isometric Knee Extension Strength (IKES)

Knee extension muscle strength was assessed using an isometric dynamometer (Isoforce GT-360; OG Wellness Technologies Co., Ltd., Okayama, Japan) [16]. Participants were seated upright on the dynamometer with the tested limb’s knee flexed at 60°. The depth, height, placement, and length of the attachment arm were adjusted for each participant to ensure optimal positioning. Stability was maintained using straps at the distal shank, mid-thigh, and pelvis. Participants were instructed to generate maximal contraction within three seconds and maintain the contraction for five seconds, with standardized verbal encouragement provided throughout the process. Each participant performed two trials, and the maximum muscle strength reached during the two trials was normalized to the participant’s body weight. The normalized values were used for data analysis.

Knee Pain

A 100-mm visual analog scale (VAS) was used to measure pain intensity in the knee scheduled for TKA.

Timed Up and Go (TUG) Test

The time it takes to stand up from a chair from a sitting position, walk three meters, turn around, and return to a sitting position was measured. This test was performed at a normal pace. An average of the two recorded trials was used in data analysis.

Statistical analysis

All statistical analyses were performed using IBM SPSS Statistics for Windows, Version 26 (Released 2018; IBM Corp., Armonk, New York, United States). A significance level of p < 0.05 was set for all tests. Descriptive statistics were calculated as the mean ± standard deviation for continuous variables. Normality of continuous variables was determined using the Shapiro-Wilk test. All data were compared between supinated foot posture and neutral foot posture using Independent t-tests for parametric continuous variables and Mann-Whitney U tests for nonparametric continuous variables.

## Results

Comparing the neutral (n = 33 (53%)) and supinated (n = 29 (47%)) foot posture groups revealed several significant differences in biomechanical and functional parameters (Tables 1-3).

**Table 1:** Patient demographic data by groups SD: standard deviation; BMI: body mass index; FTA: femorotibial angle; TUG Test: Timed Up and Go Test Values ​​are expressed as n (%). Items with t-values ​​were calculated using the independent t-tests. Items with Z were calculated using the Mann-Whitney U test, with a large effect size (r) ≥ 0.50, a medium effect size (r) = 0.30-0.49, a small effect size (r) = 0.10-0.29, and a negligible effect size (r) ≤ 0.09

	Neutral n = 33 (53)		Supinated n = 29 (47)		p-value	t-value	z	Effect size (r)
	Mean	SD	Mean	SD				
Age (years old)	77.18	4.14	74.90	5.98	0.073		-1.793	0.23
Body height (cm)	152.30	6.32	153.17	5.73	0.575	-0.564		0.07
Body weight (kg)	56.78	9.52	60.90	10.39	0.069		1.815	0.23
BMI (kg/m^2^)	24.56	4.38	25.85	3.24	0.092		1.686	0.21
FTA (degree)	182.34	5.20	184.68	4.47	0.046		1.993	0.26
TUG (seconds)	11.14	3.47	12.22	4.11	0.169		1.376	0.18

**Table 2:** Foot Posture Index-6 by groups SD: standard deviation; FPI-6: Foot Posture Index-6 Values ​​are expressed as n (%). All statistical analyses were calculated using the Mann-Whitney U test, with a large effect size (r) ≥ 0.50, a medium effect size (r) = 0.30-0.49, a small effect size (r) = 0.10-0.29, and a negligible effect size (r) ≤ 0.09. Each criterion was individually scored from -2 (indicating highly supinated posture) to +2 (indicating highly pronated posture). The sum of these six items was then used to determine overall foot posture classification as follows: pronated foot (+6 to +12), neutral foot (0 to +5), or supinated foot (-1 to -12)

	Neutral n = 33 (53)		Supinated n = 29 (47)		p-value	z	Effect size (r)
	Mean	SD	Mean	SD			
FPI-6 score (total points)	2.18	1.81	-3.03	2.24	<0.01	-6.786	0.86
FPI-6 item 1 (points)	1.09	0.95	0.59	0.98	0.023	-2.281	0.29
FPI-6 item 2 (points)	-0.64	0.60	-1.10	0.82	0.01	-2.585	0.33
FPI-6 item 3 (points)	-0.58	1.06	-1.69	0.66	<0.01	-4.586	0.58
FPI-6 item 4 (points)	1.00	0.79	-0.52	1.02	<0.01	-5.189	0.66
FPI-6 item 5 (points)	1.39	0.56	0.55	0.78	<0.01	-4.217	0.54
FPI-6 item 6 (points)	-0.09	1.07	-0.86	1.03	<0.01	-2.747	0.35

**Table 3:** Knee and ankle function by groups. SD: standard deviation; ROM: range of motion; IKES: isometric knee extension strength; BW: body weight; VAS: visual analog scale Values ​​are expressed as n (%). Items with t-values ​​were calculated using the independent t-tests. Items with Z were calculated using the Mann-Whitney U test, with a large effect size (r) ≥ 0.50, a medium effect size (r) = 0.30-0.49, a small effect size (r) = 0.10-0.29, and a negligible effect size (r) ≤ 0.09

	Neutral n = 33 (53)		Supinated n = 29 (47)		p-value	t-value	z	Effect size (r)
	Mean	SD	Mean	SD				
Knee flexion ROM (degree)	134.39	11.64	122.93	15.44	<0.01	3.324		0.39
Knee extension ROM (degree)	-6.06	6.47	-6.21	6.50	0.947		-0.066	0.01
Standing knee extension angle (degree)	-8.91	5.87	-10.1	7.01	0.506		-0.666	0.09
IKES 60 degree/BW (Nm/kg)	1.07	0.32	0.86	0.30	0.011	2.630		0.32
Knee pain VAS (mm)	51.42	30.10	52.86	29.3	0.850	-0.190		0.02
Ankle Dorsiflexion ROM (degree)	7.67	5.83	4.62	4.67	0.016		-2.399	0.31
Toe grip strength/BW (kg/kg)	0.18	0.07	0.13	0.06	<0.01	2.958		0.36

The weight-standardized TGS was significantly lower in the supinated foot posture group (p < 0.01). The ankle dorsiflexion ROM was also significantly lower in the supinated foot posture group (p = 0.016). Regarding knee joint function, the knee flexion ROM was significantly lower in the supinated foot posture group compared to the neutral foot posture group (p < 0.01), but there was no significant difference in knee extension ROM and standing knee extension angle. The IKES at 60° was significantly lower in the supinated foot posture group (p = 0.011). The FTA was significantly greater in the supinated foot posture group (p = 0.046). However, there were no significant differences between groups in TUG test performance and knee pain VAS score.

## Discussion

This study examined differences in knee and foot function between KOA patients with supinated and neutral foot postures. The supinated foot posture group showed significantly lower TGS, ankle dorsiflexion, and knee flexion ROM were significantly decreased, as was knee extension strength at 60° flexion. FTA was significantly larger in the supinated foot posture group. While knee pain and TUG did not differ significantly, these impairments suggest that foot posture influences lower limb biomechanics.

TGS

Our study demonstrated that patients with supinated foot posture exhibited significantly lower TGS compared to those with neutral foot posture. This result is consistent with existing literature highlighting the important role of TGS in maintaining lower limb stability and functional mobility in patients with KOA.

Consistent with our results, Uritani et al. highlighted a significant association between decreased TGS and greater KOA severity, supporting the notion that impaired toe strength contributes directly to functional decline [12]. Additionally, Saito et al. demonstrated altered foot pressure distributions in patients with medial KOA, characterized by decreased pressure on the hallux, correlating with impaired knee and foot function [17]. These abnormal pressure patterns likely reflect reduced toe gripping efficiency, a condition evident in our supinated foot cohort.

Collectively, these findings [12,17] emphasize the complex relationship between foot posture, intrinsic toe muscle strength, and knee function. Our study supports the view that supinated foot posture worsens lower limb functional impairment through reduced TGS.

Ankle dorsiflexion ROM

Our study demonstrated that patients exhibiting a supinated foot posture had significantly lower ankle dorsiflexion ROM compared to those with a neutral foot posture. The relationship between foot posture and lower limb biomechanics has been extensively documented in prior research, lending support to our findings. Tan et al. reported that altered foot postures, including supinated alignment, are closely linked to reduced ankle dorsiflexion, which consequently contributes to increased knee pain and compromised functional mobility, particularly during weight-bearing activities such as stair ascent and sit-to-stand transitions [18]. Similarly, Johanson et al. demonstrated that subtalar joint positioning significantly affects ankle dorsiflexion during gastrocnemius stretching, suggesting that foot posture directly impacts ankle flexibility [19].

However, not all studies support this direct link between foot posture and knee loading. For example, McCarthy et al. did not identify significant associations between foot posture variations and knee loading characteristics during gait analysis [20]. The discrepancy between our results and those of McCarthy et al. could stem from methodological and contextual differences, notably in patient characteristics, osteoarthritis severity, and assessment conditions. Specifically, McCarthy et al. studied individuals with early-stage KOA and mild symptomatology, contrasting with our population of advanced KOA patients scheduled for TKA. Additionally, differences in assessment methodologies, such as static versus dynamic foot posture evaluation, could further contribute to inconsistent findings across studies.

FTA

In this study, patients with a supinated foot posture exhibited significantly larger FTA compared to those with neutral foot posture, suggesting a potential association between foot supination and increased varus knee alignment in individuals with KOA. This finding is consistent with prior reports on the biomechanical impact of foot posture on proximal joint alignment. Al-Bayati et al. reported that foot supination was significantly associated with radiographic severity of KOA, including increased condylar plateau angle and medial joint space narrowing [6]. They proposed that supination induces a medial shift in the ground reaction force vector, thereby elevating the knee adduction moment, a recognized contributor to the progression of varus deformity. The present result, demonstrating greater FTA in patients with supinated feet, supports this biomechanical rationale.

Further reinforcing this interpretation, Burssens et al. performed a coronal alignment analysis using weight-bearing computed tomography and demonstrated a significant association between hindfoot varus and mechanical tibiofemoral varus angle in patients with tibiotalar arthritis [21]. Their findings highlight the coronal interdependence of distal and proximal joint alignment and suggest that foot morphology may influence knee joint loading through compensatory mechanisms.

However, it is important to note that the compensatory relationship between foot and knee alignment remains complex. Norton et al., in a radiographic study of patients with advanced KOA, reported a significant association between hindfoot valgus and knee varus alignment, suggesting that hindfoot valgus may develop as a compensatory mechanism in response to varus deformity at the knee [22]. It should be noted that there is a methodological difference between the present study and that of Norton et al. While Norton’s analysis was limited to radiographic assessment of hindfoot alignment, our study employed the FPI-6, a comprehensive clinical tool that evaluates the position of the entire foot, including both forefoot and hindfoot contributions. Therefore, our results may capture a broader spectrum of foot alignment characteristics and their influence on proximal joint morphology. Taken together, these findings highlight the value of whole-foot assessment in understanding the complex interdependence between foot posture and knee joint alignment in patients with KOA.

TUG and knee pain

In the present study, we observed no significant differences between patients with supinated and neutral foot postures regarding functional mobility as assessed by the TUG test or knee pain severity.

Consistent with our findings, Mohd Said et al. reported no significant differences in TUG performance when comparing older adults with supinated and neutral foot postures [23]. Although neutral feet are generally considered to provide a broader base of support and thus greater stability, variations in foot alignment did not appear to substantially impact functional mobility as measured by the TUG test.

In contrast to the results of this study, Akaltun et al. and Al-Bayati et al. reported that patients with supinated foot posture had significantly more pain than those patients with neutral foot posture, as measured by the Western Ontario and McMaster Universities Osteoarthritis Index (WOMAC) pain subscale [6,8]. Furthermore, Akaltun et al. also found that patients with supinated foot posture had higher VAS pain scores [8]. One reason for these differences may be the difference in patient populations. In both studies, approximately 60% of participants had a low KL grade and a mean age in their 50s or 60s. In contrast, the present study included participants with high KL grade KOA and a mean age in their 70s. These results suggest that pain severity may be more strongly influenced by disease duration and radiographic severity than by foot posture alone.

Knee flexion ROM and IKES

Our study demonstrated that patients with KOA and supinated foot posture exhibited significantly reduced knee flexion ROM and decreased IKES compared to those with neutral foot posture. These biomechanical differences likely reflect anatomical and functional adaptations occurring within the lower limb kinetic chain, influenced by altered foot alignment.

The observed reduction in knee flexion ROM among patients with supinated feet may be attributed to increased tension and stiffness of lateral lower limb structures, notably the iliotibial tract (ITT) and tensor fascia latae (TFL). Supinated foot posture, characterized by excessive subtalar inversion, can induce lateral soft tissue tightness, resulting in mechanical constraints on knee flexion through increased lateral compartment stiffness and reduced joint compliance. Such adaptations potentially exacerbate lateral joint loading, ultimately restricting knee joint mobility. Supporting these interpretations, Liu et al. highlighted that altered muscle activation patterns, such as increased vastus lateralis (VL) activation and biceps femoris involvement, can accompany structural foot deviations, reinforcing the lateral knee compartment's stiffness and potentially limiting ROM​ [24].

The reduction in knee extension strength observed in our supinated foot posture group aligns with indirect evidence from previous studies, suggesting a functional interplay between foot posture and quadriceps performance. Golightly et al. reported that supinated foot posture may alter knee biomechanics, potentially increasing lateral compartment loading and promoting compensatory VL muscle hyperactivity [25]. This imbalance could impair optimal activation of knee extensors, reducing their mechanical effectiveness and resulting in decreased IKES.

Therefore, our findings underscore the biomechanical complexity linking supinated foot posture with decreased knee flexion ROM and impaired IKES. While anatomical factors such as increased ITT and TFL tension and compensatory VL muscle activation provide plausible mechanisms, the nuanced interactions of these elements require further detailed biomechanical and electromyographic investigations.

Summary

In summary, our findings highlight critical biomechanical impairments associated with supinated foot posture in patients with KOA, emphasizing its potential role in altered knee joint loading patterns and functional deterioration. Recognizing these biomechanical relationships can guide clinicians toward more targeted interventions, such as foot-specific rehabilitation exercises, aimed at relieving abnormal loading patterns and potentially slowing disease progression. Future research employing longitudinal designs and dynamic biomechanical assessments is necessary to further clarify the role of foot posture in KOA progression and to establish evidence-based intervention strategies.

Limitations

This study has several limitations that should be considered when interpreting the results. Participants were limited to female patients with advanced KOA scheduled for TKA, excluding males, individuals with less severe KOA, and those with pronated foot posture. The absence of a pronated foot group limits the comprehensive understanding and generalizability of the results across all foot posture types commonly seen in clinical practice.

Importantly, pronated foot posture was excluded from this study for several reasons. First, the number of participants classified with a pronated foot posture was insufficient to allow for meaningful statistical analysis. Second, the primary objective of this research was to explore functional and biomechanical differences between supinated and neutral foot postures, given the clinical need to develop targeted interventions for foot supination, such as lateral wedge insoles or dorsiflexion stretching programs for KOA management. Therefore, this study focused on comparing supinated and neutral foot posture groups.

FPI-6 and ankle dorsiflexion were evaluated under static conditions. Dynamic assessments during weight-bearing tasks or gait analysis might provide more accurate insights into functional impairments and compensatory mechanisms.

While age, BMI, and knee severity were considered, other relevant factors, such as physical activity levels, neuromuscular coordination, or proprioceptive function, were not measured. These could influence muscle strength, foot posture, and knee biomechanics.

This study did not evaluate changes in foot posture, TGS, or knee function after TKA. The effects of surgical intervention and postoperative rehabilitation on these parameters are unknown. Future studies should incorporate preoperative and postoperative comparisons. Because measurements of hallux valgus were not taken, it is not possible to say with certainty whether the decrease in toe gripping strength was due to the effects of hallux valgus or to knee alignment or function. Future research addressing these limitations could yield more comprehensive insights and enhance clinical relevance in KOA rehabilitation strategies.

## Conclusions

This study demonstrated that patients with KOA who exhibit a supinated foot posture have significantly lower knee flexion, reduced ankle dorsiflexion ROM, decreased IKES, weaker TGS, and larger FTA compared to patients with a neutral foot posture. However, no significant differences were identified in the TUG test or knee pain severity between the two groups. Our findings provide valuable insights into potential biomechanical associations between foot posture and lower limb function in KOA patients.

## References

[1] Levinger P, Menz HB, Morrow AD, Bartlett JR, Feller JA, Bergman NR (2013). Relationship between foot function and medial knee joint loading in people with medial compartment knee osteoarthritis. J Foot Ankle Res.

[2] Parekh Sanket, Mehta Jigar, Vaghela Nirav, Ganjiwale Deepak (2019). Association between the lower extremity biomechanical factors with osteoarthritis of knee. J Med Soc.

[3] Wang Y, Chen Z, Wu Z (2023). Reliability of foot posture index (FPI-6) for evaluating foot posture in patients with knee osteoarthritis. Front Bioeng Biotechnol.

[4] Levinger P, Menz HB, Fotoohabadi MR, Feller JA, Bartlett JR, Bergman NR (2010). Foot posture in people with medial compartment knee osteoarthritis. J Foot Ankle Res.

[5] Mei Q, Kim HK, Xiang L (2022). Toward improved understanding of foot shape, foot posture, and foot biomechanics during running: A narrative review. Front Physiol.

[6] Al-Bayati Z, Coskun Benlidayi I, Gokcen N (2018). Posture of the foot: don't keep it out of sight, out of mind in knee osteoarthritis. Gait Posture.

[7] Gatt A, Chockalingam N, Chevalier TL (2011). Sagittal plane kinematics of the foot during passive ankle dorsiflexion. Prosthet Orthot Int.

[8] Akaltun MS, Koçyiğit BF (2021). Assessment of foot posture and related factors in patients with knee osteoarthritis. Arch Rheumatol.

[9] Karataş L, Utkan Karasu A (2024). Association of medial longitudinal arch height and stiffness with lower extremity alignment, pain, and disease severity in knee osteoarthritis: a cross-sectional study. Arch Rheumatol.

[10] Chen Z, Ye X, Shen Z (2021). Comparison of the asymmetries in foot posture and properties of gastrocnemius muscle and Achilles tendon between patients with unilateral and bilateral knee osteoarthritis. Front Bioeng Biotechnol.

[11] Wang Y, Zhang P, Chen G, Jiang T, Zou Y (2024). Comparison of the asymmetries in foot posture, gait and plantar pressure between patients with unilateral and bilateral knee osteoarthritis based on a cross-sectional study. Sci Rep.

[12] Uritani D, Fukumoto T, Myodo T, Fujikawa K, Usui M, Tatara D (2017). The association between toe grip strength and osteoarthritis of the knee in Japanese women: a multicenter cross-sectional study. PLoS One.

[13] Uritani D, Fukumoto T, Matsumoto D, Shima M (2014). Reference values for toe grip strength among Japanese adults aged 20 to 79 years: a cross-sectional study. J Foot Ankle Res.

[14] Redmond AC, Crosbie J, Ouvrier RA (2006). Development and validation of a novel rating system for scoring standing foot posture: the Foot Posture Index. Clin Biomech (Bristol).

[15] Uritani D, Fukumoto T, Matsumoto D (2012). Intrarater and interrater reliabilities for a toe grip dynamometer. J Phys Ther Sci.

[16] Fujita R, Ota S, Yamamoto Y (2024). Factors associated with physical activity following total knee arthroplasty for knee osteoarthritis: a longitudinal study. BMC Musculoskelet Disord.

[17] Saito I, Okada K, Nishi T (2013). Foot pressure pattern and its correlation with knee range of motion limitations for individuals with medial knee osteoarthritis. Arch Phys Med Rehabil.

[18] Tan JM, Crossley KM, Munteanu SE (2020). Associations of foot and ankle characteristics with knee symptoms and function in individuals with patellofemoral osteoarthritis. J Foot Ankle Res.

[19] Johanson MA, DeArment A, Hines K, Riley E, Martin M, Thomas J, Geist K (2014). The effect of subtalar joint position on dorsiflexion of the ankle/rearfoot versus midfoot/forefoot during gastrocnemius stretching. Foot Ankle Int.

[20] McCarthy I, Hodgins D, Mor A, Elbaz A, Segal G (2013). Analysis of knee flexion characteristics and how they alter with the onset of knee osteoarthritis: a case control study. BMC Musculoskelet Disord.

[21] Burssens AB, Buedts K, Barg A, Vluggen E, Demey P, Saltzman CL, Victor JM (2020). Is lower-limb alignment associated with hindfoot deformity in the coronal plane? A weightbearing CT analysis. Clin Orthop Relat Res.

[22] Norton AA, Callaghan JJ, Amendola A, Phisitkul P, Wongsak S, Liu SS, Fruehling-Wall C (2015). Correlation of knee and hindfoot deformities in advanced knee OA: compensatory hindfoot alignment and where it occurs. Clin Orthop Relat Res.

[23] Mohd Said A, Manaf H, Bukry SA, Justine M (2015). Mobility and balance and their correlation with physiological factors in elderly with different foot postures. Biomed Res Int.

[24] Liu S, Du Z, Song L, Liu H, Tee CA, Liu H, Liu Y (2025). Factors, characteristics and influences of the changes of muscle activation patterns for patients with knee osteoarthritis: a review. J Orthop Surg Res.

[25] Golightly YM, Dufour AB, Hannan MT, Hillstrom HJ, Katz PP, Jordan JM (2016). Leg muscle mass and foot symptoms, structure, and function: the Johnston County osteoarthritis project. J Gerontol A Biol Sci Med Sci.

